# Chinese Propolis Exerts Anti-Proliferation Effects in Human Melanoma Cells by Targeting NLRP1 Inflammatory Pathway, Inducing Apoptosis, Cell Cycle Arrest, and Autophagy

**DOI:** 10.3390/nu10091170

**Published:** 2018-08-26

**Authors:** Yufei Zheng, Yuqi Wu, Xi Chen, Xiasen Jiang, Kai Wang, Fuliang Hu

**Affiliations:** 1 College of Animal Sciences, Zhejiang University, Hangzhou 310058, China; iriszheng92@gmail.com (Y.Z.); 11317024@zju.edu.cn (Y.W.); 21717031@zju.edu.cn (X.C.); jxsen@zju.edu.cn (X.J.); 2 Institute of Apicultural Research, Chinese Academy of Agricultural Sciences, Beijing 100093, China; kaiwang628@gmail.com

**Keywords:** Chinese propolis, anti-melanoma activity, apoptosis, anti-inflammatory activity, autophagy induction, cell cycle

## Abstract

Melanoma is a malignant tumor that begins in the melanocyte and has the highest mortality rate among all cutaneous tumors. Chinese propolis (CP) has been shown to have a potent antitumor effect against various cancers. In this study, we uncovered the combined effects of antiproliferation and anti-inflammation of CP on suppressing the progression of human melanoma cell line A375. We evaluated the alterations of protein expression after CP treatment by Western blot. After CP treatment, A375 cells underwent intrinsic apoptosis and cell cycle arrest. Furthermore, we found that CP suppressed inflammation in A375 cells. NLRP1 (NLR family pyrin domain containing 1), confirmed as a proinflammatory protein in melanoma progression, was downregulated significantly by CP, as were the NLRP1-related caspase activation and recruitment domains (CARD) proteins, including caspase-1 and caspase-4. Additionally, decreasing mRNA levels of *IL-1α*, *IL-1β*, and *IL-18* further proved the negative regulation of CP on the melanoma inflammatory environment. We also discovered that CP induced autophagy in A375 cells. Interestingly, inhibiting autophagy in CP-treated cells diminished its antitumor effect, suggesting that the autophagy was attributed to CP-induced apoptosis. Collectively, CP is a promising candidate for drug development for melanoma therapy.

## 1. Introduction

Melanoma is the most rapidly increasing malignant skin disease, and is characterized by high lethality and frequent metastasis [1,2]. The incidence of melanoma has risen faster than any other solid tumor [3]. According to a WHO report, there were 3.1 million new diagnoses of melanoma and 59,800 deaths due to the disease worldwide in 2015 [4]. UV exposure, genetic factors, DNA damage, and immune conditions are all recognized as contributors to melanoma development [1,2]. Meanwhile, Reactive oxygen species (ROS), damaged DNA and altered cell homeostasis are also known to interact with inflammasomes [5,6]. Following stimulation, such as UV light, keratinocytes, melanocytes, and Langerhans cells will secrete pro-inflammatory cytokines which build an inflammatory microenvironment allowing tumorigenesis and metastasis [7]. Therefore, researchers have linked melanoma with inflammation and confirmed that inflammation is chronically activated in human melanoma, acting as a promoter [8,9,10]. Different cytokines, like IL-6, TNF-α, IFN-γ, IL-10, and TGF-β1, have been reported to play a role in the progression and immunosurveillance escape of melanoma [11]. In addition, IL-1 was found to be expressed constitutively in most human melanoma and function to support molecular pathways of inflammation and facilitate the tumor growth [12]. Nucleotide-binding domain and leucine-rich repeat-containing receptors (NLRs) are the most important and well-studied inflammation signaling cascade initiators, and they also play a fundamental role in the regulation of proliferation, cell survival and death, ROS generation and angiogenesis [13,14]. A recent report has pointed out that NLRP-1 is expressed constantly in human melanoma [10]. The knockdown of *NLRP-1* resulted in reduced IL-1β production and secretion, which led to the reduction of tumor proliferation in vivo and in vitro [10]. Thus, the NLR inflammatory pathways can be a potential target for melanoma therapy [15].

Autophagic cell death is considered to be one type of programmed cell death and interacts closely with apoptosis [16]. Cells undergoing autophagy can promote either go to survival or death, depending on which role autophagy plays in the response to the external stimuli [17,18]. The activation of autophagy depends on Atg5/Atg7, which is associated with the truncation and lipidation of LC3, and beclin1 is indispensable for Atg5/Atg7-dependent autophagy. Beclin1 has a central role in autophagy and accumulates when the cell is under stress. It interacts with NLRs, such as NLRC4, NLRP3, NLRP1 and can be suppressed by Bcl-2 and Bcl-XL [19,20,21]. Therefore, beclin1 serves as a linkage between autophagy and inflammation, which is considered to be another way to regulate autophagy. On the other hand, growing evidence has shown that autophagy induced by antitumor agents enhanced their cytotoxicity against cancers, implying the therapeutic potential of autophagy in cancers [22,23,24].

The cell cycle is considered to be another target for restricting tumor proliferation [25]. Checkpoint signaling in the cell cycle also results in the activation of pathways leading to programmed cell death if cellular damage cannot be properly repaired [26]. In regard to cancer therapy, cell cycle deregulation sensitizes tumor cells in response to antitumor agents, and there is considerable evidence that the G2 phase delay can affect the survival of cancer cells [25]. The progression from G2 to the M phase is regulated by the cyclinB/cdk1 complex and can be interrupted by ATM and ATR [27]. In addition to the cyclinB/cdk1 complex, p21 also can disrupt the proliferating cell nuclear antigen (PCNA) and cdc25c to induce G2 cell-cycle arrest [25].

Nowadays, growing evidence have shown that bee hive derivatives have the potential for development in medical therapy. For instance, royal jelly and its fractions have been proven to have an antiproliferative effect on human neuroblastoma cells [28] and can be used as a functional food [29]. Another noticeable bee product is propolis. Propolis is a resinous product collected by the honey bee from plants and possesses a broad spectrum of biological activities [30,31], and its use as a folk medicine can be traced back to ancient China. Research has been carried out to examine the antioxidant and anti-inflammatory effects of the combination of honey and propolis [32]. The antitumor effect of Chinese propolis (CP), such as eliciting apoptosis and cell cycle arrest in vivo and in vitro, has been reported in different cancer models including breast cancer, colon cancer, etc. [33,34,35,36]. However, its application in melanoma therapy has not been observed yet. Here, for the first time, we presented the potential pharmacological use of CP for melanoma proliferation suppression via inducing apoptosis, S-G2/M phase arrest, autophagy, and inhibiting the inflammatory microenvironment in melanoma in vitro.

## 2. Materials and Methods

### 2.1. Reagents

Fetal bovine serum (FBS) was purchased from Gibco (New York, NY, USA). Chloroquine (CQ) and Fluorouracil (5-FU) were purchased from Sigma (St Louis, MO, USA). Propidium iodide (PI) and dimethyl sulfoxide (DMSO) were purchased from Sangon Biotechnology. Co. Ltd. (Shanghai, China). The primary antibodies against β-tublin, MMP-2, cyclinB1, p21, cdk-2, cdc-2, NLRP3, caspase-1, caspase-2, caspase-3, caspase-8, caspase-9, PARP, Bcl-2 and Bax along with anti-rabbit secondary antibodies (ab191866), were purchased from Abcam (Cambridge, UK). NLRP1, Atg12, p-chk1, LC-3 and MMP-9 antibodies were purchased from Cell Signaling Technology (Danvers, MA, USA). Caspase-4, p62 and beclin1 antibodies were purchased from ProteintechGroup (Rosemont, PA, USA).

### 2.2. Cell Culture

HEK-293 and A375 cells were gifted by Zhejiang University of Traditional Chinese Medicine and authenticated by STR analysis. Cells were cultured in DMEM supplemented with 10% heat-inactivated FBS (Gibco) in 10 cm × 10 cm culture dishes at 37 °C in a humidified atmosphere of 5% CO_2_. Cells were grown to confluence prior to drug administration.

### 2.3. Extraction of Chinese Propolis (CP)

The raw Chinese propolis was obtained from colonies of honey bee, *Apis mellifera* L., in Zhejiang province, and the main plant origin was poplar. Raw propolis was extracted by 95% (*v*/*v*) ethanol and sonicated at 40 °C for 3 h. The raw propolis was extracted three times. The combined supernatant was filtered through a paper filter to remove the residues, and the solvent was evaporated in a rotary evaporator under a reduced pressure at 50 °C to evaporate the ethanol until reaching a constant weight. CP was dissolved in ethanol (95%) to get 50 mg/mL stock solutions. Samples were stored at −20 °C for further use, and the 95% (*v/v*) ethanol was used as a vehicle control.

### 2.4. Liquid Chromatography−Mass Spectrometry (LC–MS) Analysis

The major compounds determined in CP we used were similar to previous studies [33,37]. A Waters UPLC (Waters Corp., Milford, MA, USA) was used for the analysis, and an Agilent ZORBAX-SB C18 column (100 mm length × 4.6 mm I.D. × 1.8 µm particle size) was used in all the chromatographic experiments. The mobile phases used were 0.1% formic acid/water (mobile phase A) and 0.1% formic acid/acetonitrile (mobile phase B), and the linear gradient programs were set as follows: 0/5, 2/5, 25/50, 33/95 (min/mobile phase B%). The sample injection volume was 10 μL. The column oven temperature was held at 30 °C, the flow rate was 0.8 mL min^−1^, and the UV detector was set at 254 nm. The mass spectrometry used in this research was the AB TripleTOF 5600 plus System (AB SCIEX, Framingham, MA, USA). The optimal MS conditions were set as follows: the scan range was 100–2000 m/z in negative ion mode, the source voltage was −4.5 kV, and the source temperature was 550 °C. The pressure of gas 1 (Air) and gas 2 (Air) were set to 50 psi, and the pressure of the curtain gas (N_2_) was set to 35 psi. The maximum allowed error was set to ±5 ppm. The declustering potential (DP) was 100 V, and collision energy (CE) was 10 V. The IDA-based auto-MS2 was performed on the eight most intense metabolite ions in a cycle of full scan cycle (1 s). The scan ranges of m/z of precursor ions and product ions were set as 100–2000 Da and 50–2000 Da, respectively. The CE voltage was set at −20, −40, and −60 V in the negative ESI mode. Ion release delay (IRD) was set at 67 and ion release width (IRW) was at 25. The exact mass calibration was performed automatically before each analysis by employing the Automated Calibration Delivery System.

### 2.5. Cell Viability Assay

Cell viability was measured using the CCK-8 kit (cell counting kit-8) (Dojindo, Kumamoto, Japan) according to the manufacturer’s instructions. Cells (2 × 10^4^) were seeded onto 96-well cell culture plates and were cultured in varying concentrations of CP after 24 h of incubation. After 48 h of treatment, the cells were incubated with 10 μL of CCK-8 at 37 °C for 2 h. The optical density was measured at 450 nm using a microplate reader (Bio-Rad Model550, Hercules, CA, USA).

### 2.6. Cell Apoptosis Assay

A375 cells were treated with CP for 48 h and were then harvested, washed twice with phosphate-buffered saline (PBS), and centrifuged. Cells (1 × 10^5^) were seeded onto 6-well cell culture plates and treated with Annexin V-FITC and PI using the Apoptosis Detection Kit (Dojindo, Kumamoto, Japan) according to the manufacturer’s protocol after 48 h treatment of CP. Annexin V-FITC and PI binding were analyzed by flow cytometry on FACSCalibur with CellQuest (Becton Dickinson, Franklin Lakes, NJ, USA).

### 2.7. Cell Cycle Assay

A375 cells were seeded at a density of 3 × 10^5^ in 60 mm culture dishes and grown to 50% confluence. Subsequently, the cells were cultured in serum-free medium for 24 h and then treated with 6–50 μg/mL of CP for 12–48 h in complete medium. After treatment, cells were collected and processed according to the Cell Cycle and Apoptosis Analysis kit purchased from Beyotime Biotechnology (Shanghai, China). The results were analyzed by software Modfit 3.0.

### 2.8. Western Blotting

A density of 1 × 10^5^ A375 cells were seeded onto 6-well plates. After 24 h, cells were treated with varying concentrations of CP for 12 h, 24 h and 48 h. Then cells were washed twice immediately with pre-chilled PBS on ice. The cytoplasmic proteins were lysed using RIPA mixed with protease inhibitors and phosphatase inhibitors (Roche, Basel, Switzerland). The protein concentration was measured by the BCA protein assay kit (Fudebio, Hangzhou, China). The protocol we used was that same with our previous publication [38].

### 2.9. RNA Isolation and Quantitative Real-Time Polymerase Chain Reaction (qRT-PCR)

Total RNA from A375 cells was extracted using commercial RNA extraction kits (Aidlab Biotechnologies Co. Ltd., Beijing, China) according to the manufacturer’s protocols. The concentration of RNA in the samples was measured by NanoDrop spectrophotometer (ND-2000, NanoDrop Technologies, Wilmington, DE, USA) and stored at −80 °C until further use. For cDNA synthesis, 1 μg of RNA was used with the Prime Script RT Master Mix (TaKaRa, Dalian, China). qRT-PCR was performed using StepOne plus (Applied Biosystems, Carlsbad, CA, USA) with a SYBR Premix Ex Taq (TaKaRa, Dalian, China) via a standard two-step PCR reaction. Specificity was confirmed by melting curve analysis. The housekeeping gene *GAPDH* was used to normalize the expression of the other target genes, and the results were expressed as 2^−ΔΔCt^. The primers we used are provided in Table 1.

### 2.10. Wound Healing Assay

For the in vitro wound healing assay, we used culture insert 2-well (ibidi GmbH, Planegg, Germany) according to the manufacture’s protocol. After 24 h the culture-insert was removed, leaving a cell-free gap of approximately 500 mm in width. Then the cells were treated with a vehicle (DMEM) or nonfatal concentrations of CP. The migration rate was documented every 12 h using a microscope.

### 2.11. ROS Determination

To evaluate the levels of intracellular ROS, A375 cells were treated with the ROS-sensitive dye, DCFH2–DA, for 30 min at 37 °C under 5% CO_2_. The cells were then harvested and washed three times with PBS, after which the intensity of fluorescence was measured by flow cytometry using an excitation wavelength of 488 nm and an emission wavelength of 525 nm (Becton Dickinson, Franklin Lakes, NJ, USA).

### 2.12. Statistical Analysis

For each assay, at least three experiments were conducted. Results are expressed as means ± SE. Comparisons between the control and each treatment were performed using Student’s unpaired *t*-test (* *p* < 0.05; ** *p* < 0.005; *** *p* < 0.001).

## 3. Results

### 3.1. Components Identified in CP

Based on previous studies, the main bioactive properties in CP were identified and quantified by LC-MS analysis (Table 2) [37,40,41], of which 3-O-Acetyl pinobanksin (73.81 mg/g), chrysin (50.99 mg/g) and pinocembrin (43.93 mg/g) were the three most abundant components in CP. Our previous study determined that pinocembrin has an antitumor effect on melanoma progression [38], and Pichichero et al. have proved that chrysin can inhibit melanoma proliferation in vitro [42]. Therefore, we assumed that CP may have cytotoxicity to melanoma cells.

### 3.2. CP Induces Mitochondria-Dependent Apoptosis in Human Melanoma Cell Line A375

**Figure 1:**
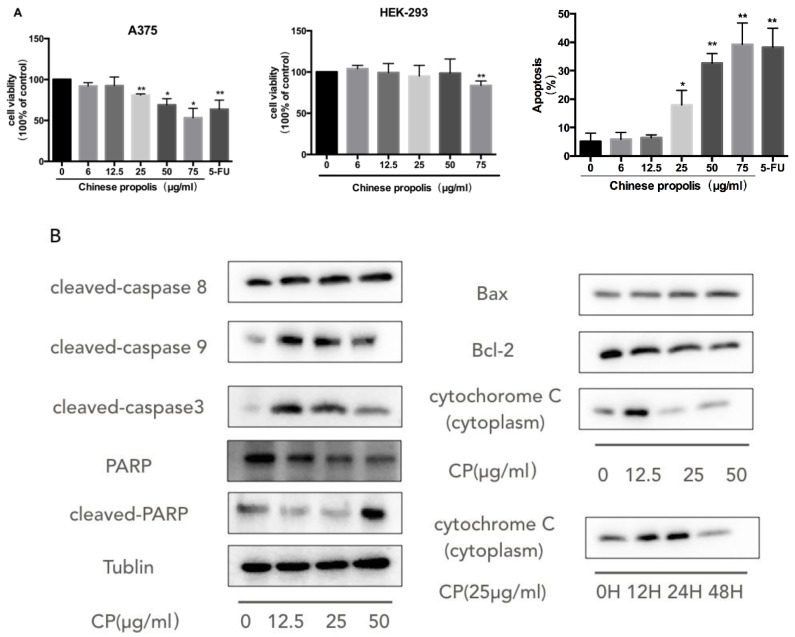
Chinese propolis (CP) exerted a pro-apoptotic effect on human melanoma cells via the mitochondria pathway. (**A**) The cells (A375, HEK-293) were cultured and incubated with 6 μg/mL, 12.5 μg/mL, 25 μg/mL, 50 μg/mL, 75 μg/mL CP and 50 μM Fluorouracil (5-FU) as a positive control for 48 h and were then analyzed using the cell counting kit-8 (CCK8) assay and Annexin-FITC/PI assay. * *p* < 0.05 and ** *p* < 0.005 for the designated treatment versus the control (vehicle); (**B**) CP induced intrinsic apoptosis and changes in the expression of Bax and Bcl-2. After treatment with CP for 48 h, the expression of apoptosis related proteins including the caspase family and mitochondria-related proteins were analyzed by Western blot.

To investigate the antiproliferation effect of CP on human melanoma cells, we treated A375 cells with varying concentrations of CP from 6 μg/mL to 75 μg/mL and then evaluated the cell viability by CCK8 assay (Figure 1A). We found that CP exerted significantly cytotoxic effect on A375 cells from 25 μg/mL in a dose-dependent manner with an IC_50_ value of approximately 112 μg/mL, while it did not cause any damage to HEK-293 cells (human embryonic kidney cell line) up to 75 μg/mL. In addition, we used the Annexin V-FITC/PI assay to assess the ratio of CP induced apoptosis in A375 cells. We found that 48 h treatment with 50 μg/mL CP resulted in 32.63% ± 1.99% apoptotic cells, as compared to 5.14% ± 2.08% in the control group (Figure 1A).

After we confirmed that CP induced apoptotic cell death in the A375 cell line, we further investigated the molecular mechanisms underlying this phenomenon. The activation of the caspase cascade including caspase-8, caspase-9, caspase-3 and PARP was observed after 48 h treatment with 12.5 μg/mL CP (Figure 1B). The role that mitochondria played in the CP-induced-caspase cascade was also analyzed. We examined the expression levels of Bcl-2 family proteins. Bcl-2 expression was diminished, while Bax was up-regulated after CP treatment. Meanwhile, the release of cytochrome c from mitochondria was observed. Its expression level in cytoplasm was elevated significantly at 12.5 μg/mL CP for 48 h or from 12 h at 25 μg/mL CP, indicating the dysfunction of mitochondria.

### 3.3. CP Induces S-G2/M Cell-Cycle Arrest in Human Melanoma Cell Line A375

**Figure 2:**
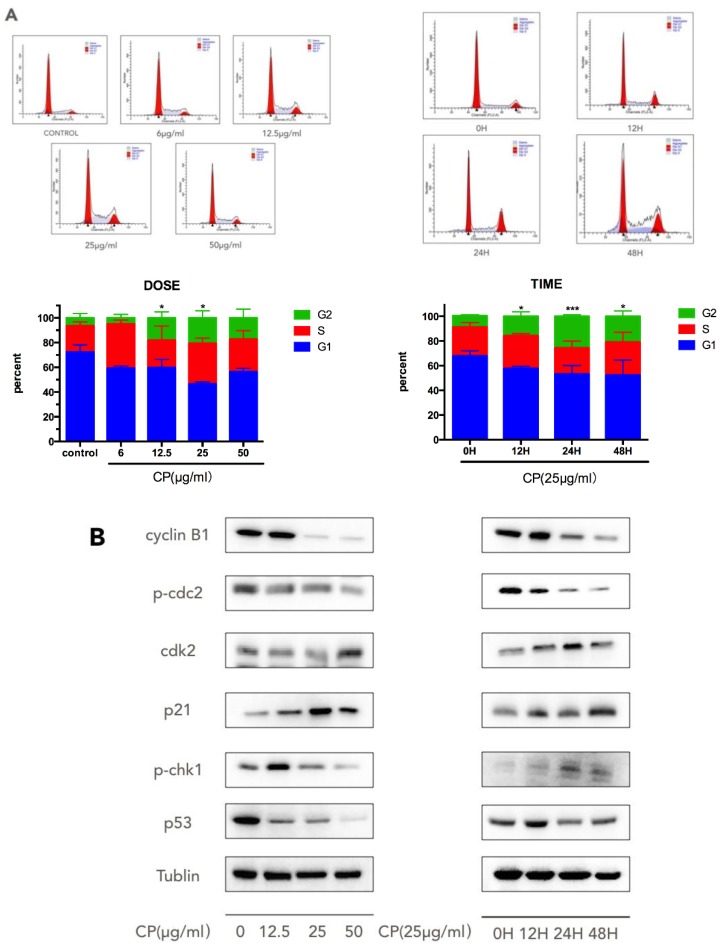
Chinese propolis (CP) triggered G2/M phase arrest in human melanoma. (**A**) The changes in the distribution of cell cycle phases after CP treatment. A375 cells were treated with 6 μg/mL, 12.5 μg/mL, 25 μg/mL and 50 μg/mL CP for 48 h or with 25 μg/mL CP for 6 h, 12 h, 24 h and 48 h. * *p* < 0.05 and *** *p* < 0.001 for the designated treatment versus the control (vehicle); (**B**) The expression of cell cycle regulation proteins was measured by Western blot.

We examined the cell-cycle distribution in A375 cells by flow cytometry after CP treatment by flow cytometry. After cells were treated with different concentrations of CP for 12, 24 and 48 h, we found that a large portion of cells were arrested at the S and G2/M phases of the cell cycle (Figure 2A). After 25 μg/mL CP treatment for 12 h, or 48 h treatment with 12.5 μg/mL CP, cells blocked at the G2/M-phase were up to 18.06% ± 2.77% and 15.82% ± 2.05%, respectively, versus 9.11% ± 0.52% and 6.48% ± 2.04% in the control group. Collectively, the present results showed that exposure to CP damaged G2-M arrest and insulted to the human melanoma cells.

Cell cycle control is implemented through a series of checkpoints [43]. To understand how CP interrupts the cell cycle progression, we next examined the expressions of the cell cycle-related proteins cyclinB1, p-cdc-2, cdk-2, p21, and p-chk-1 (Figure 2B). After 48 h treatment with CP, the expression level of cdk-2 was upregulated, while p-cdc-2 and cyclinB1 had decreased. p21 was over-expressed after treatment of CP. The alterations of protein expression were observed at 12 h, with a corresponding increase in the G2/M phase in flow cytometry results. In addition, p53 was found to be downregulated after CP treatment.

### 3.4. CP Prompts Apoptosis in A375 by Targeting NLRP1-Related Inflammatory Pathway

**Figure 3:**
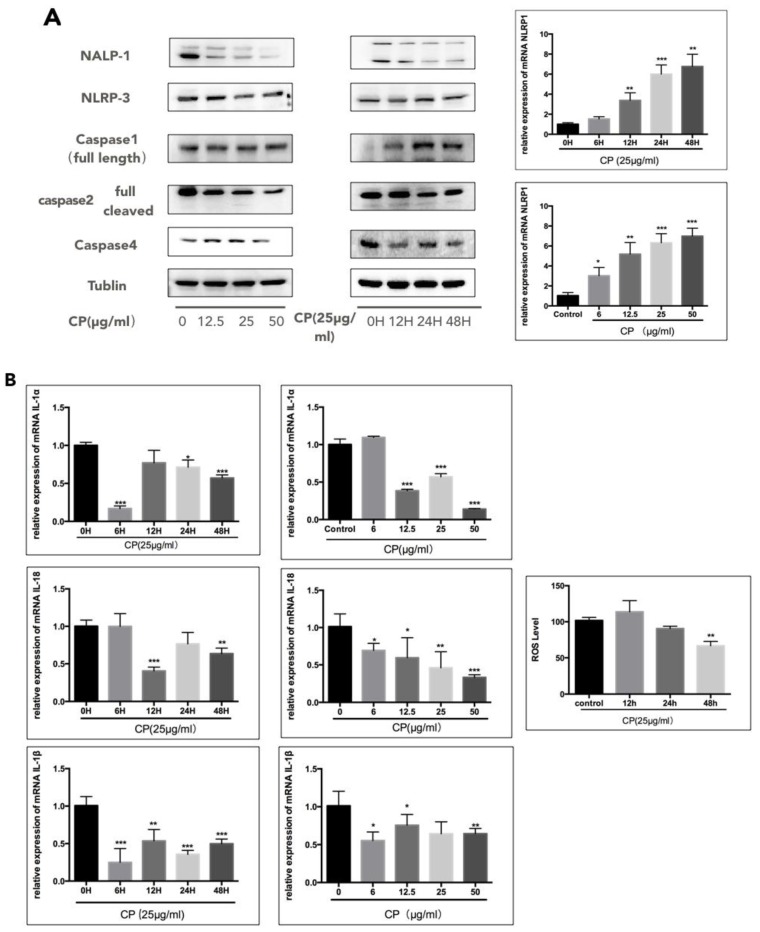

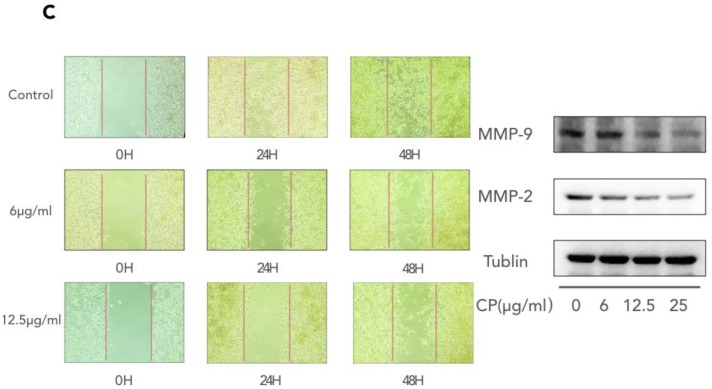
Chinese propolis (CP) suppressed melanoma progression by targeting NLRP1. (**A**) CP inhibited inflammation in human melanoma by targeting NLRP1. The NLRs and related proteins were analyzed by Western blot. In contrast to protein level, *NLRP1* mRNA level was up-regulated; (**B**) The effect of CP on the *IL-1* family was measured by qRT-PCR. The mRNA expressions of *IL-1α*, *IL-β* and *IL-18* were all decreased significantly after treatment with CP. The ROS level in human melanoma was also downregulated by CP treatment. * *p* < 0.05, ** *p* < 0.005 and *** *p* < 0.001 for the designated treatment versus the control (vehicle). Each value represents the mean ± SEM for *n* = 6; (**C**) CP suppressed migration of human melanoma. A375 cells were treated with different concentrations of CP (6 μg/mL and 12.5 μg/mL). The migration ability was determined by the wound healing assay and images were obtained by a live imaging microscope every 12 h. The activity and abundance of MMP-9 and MMP-2 of A375 cells treated with different concentration of CP (0, 6, 12.5, 25 μg/mL) for 48 h was determined by Western blot analysis.

Inflammation has long been recognized to be closely associated with various types of cancer, especially with melanoma [8,9,15]. Interestingly, we found that NLRP1 responded significantly to CP treatment, while NLRP3 expression was not influenced obviously (Figure 3A). On the other hand, the mRNA level of *NLRP1* was not consistent with the protein level and increased after CP treatment. In our study, the expression of the full-length form of caspase-1 increased, while the full-length form of caspase-2 subsequently decreased, revealing suppression of caspase-1 and activation of capase-2. Upon activation, the NLRs are known to cleave the caspase-1, which results in the activation of IL-1β [44]. Thus, we analyzed the mRNA levels of *IL-1β*, *IL-1α* and *IL-18* and found a significant decline after CP treatment, indicating the suppression of the inflammatory pathway (Figure 3B). Furthermore, the ROS level was significantly decreased after being treated with 25 μg/mL CP for 48 h (Figure 3B).

Considering that the IL-1 family has a close interaction with tumor metastasis, we performed the wound healing assay to examine if the CP treatment affects melanoma migration (Figure 3C). In line with our expectation, the migration rate of melanoma cells was slowed significantly by CP. Furthermore, in CP-treated cells, we detected the decreasing expression of MMP-2 and MMP-9, which are necessary for tumor metastasis.

### 3.5. CP Triggers Autophagy in Human Melanoma, which Enhances its Cytotoxicity to A375

**Figure 4:**
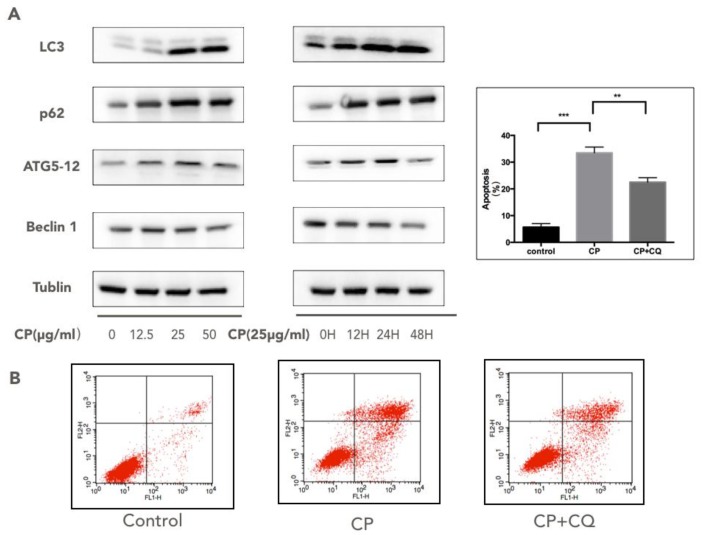
Autophagy was induced in human melanoma cells after Chinese propolis (CP) treatment, which contributed to apoptosis. (**A**) The expressions of the Atg5/Atg12 complex, Beclin1, p62 and LC-3 conversion in A375 cells after CP treatment at different doses (12.5 μg/mL, 25 μg/mL, 50 μg/mL) and times (12 h, 24 h and 48 h) were analyzed by Western blotting (**B**) The apoptosis rate induced by CP was reduced after autophagy was inhibited. A concentration of 2.5 μM chloroquine (CQ) was applied to 50 μg/mL CP-treated A375 cells to suppress autophagy. The apoptosis rate was declined significantly after co-treatment with CQ and CP in comparison with the CP-treated group. ** *p* < 0.005 and *** *p* < 0.001 for the designated treatment versus the control (vehicle). FL1-H and FL2-H represent the specific fluorescence.

Autophagy has been recognized as a tumor survival mechanism that reacts to external stimuli like antitumor drugs [16,18,45]. We examined the hallmarks of autophagy, including LC3, p62, beclin1, and Atg5/Atg12, to see if autophagy responded to CP treatment (Figure 4A). After being treated with CP for 48 h, the ratio of LC3-I/LC3-II decreased notably, while the Atg5/Atg12 complex and p62 increased, suggesting the activation of autophagy. Also, we found a down-regulation of beclin1 in CP-treated cells. To determine the role that autophagy played in CP-induced apoptosis, we applied CQ, an autophagy inhibitor, on CP-treated cells. Results showed that the apoptosis rate decreased significantly after co-treatment with CQ and CP in comparison with the CP-treated-only group (Figure 4B), indicating that autophagy partly contributed to the pro-apoptotic effect of CP on melanoma.

## 4. Discussion

Skin cancer is the third most common human malignancy, and its global incidence is rising at an alarming rate. Propolis, as an ancient folk medicine, has been recorded many times as having an antitumor effect on breast cancer, colon cancer, etc., along with its bio-components [33,46,47,48,49,50]. Furthermore, Gismondi et al. noticed that propolis is an efficient UV blocker and can be added in sunscreen to prevent UV radiation damage [51]. However, the effect of CP on melanoma still remains unknown. Here, for the first time, we presented a potential pharmacological application of CP on melanoma proliferation suppression via induction of apoptosis, S-G2/M phase arrest, autophagy, and inhibition of the inflammatory microenvironment in melanoma in vitro.

Firstly, we identified the components in the CP we used in this study. Similar to previous studies on propolis obtained from China, the main components are flavonoid and polyphenol [37,40,52]. Our results show that the CP we used was rich in 3-O-Acetyl pinobanksin, chrysin, and pinocembrin. Chrysin and pinocembrin have been noticed for their pro-apoptotic effects on melanoma cell lines [38,42]. Since the components of propolis vary according to the geographical origins and season, the functional properties and the biological functions of propolis from different locations also differ. A previous study tested Okinawa propolis (OP) on melanoma, but found that OP was only able to inhibit melanin production and had limited effect against melanoma proliferation [53].

We subsequently evaluated the anti-cancer ability of CP on human melanoma cell line A375 and discovered a dose-dependent cytotoxic effect toward A375, supported by an increase of the apoptosis rate and decline of cell viability (Figure 1). To further delineate the molecular mechanism of cell growth inhibition and apoptosis induced by CP, we investigated the protein expressions of the caspase family including the initiators caspase-8 and caspase-9, executioner caspase-3, as well as PARP. Furthermore, the Western blot results also confirmed the involvement of mitochondria dysfunction in CP-induced apoptosis, as the anti-apoptotic protein Bcl-2 expression level declined, while pro-apoptotic protein Bax increased at the same time. The release of cytochrome c from mitochondria to cytoplasm also provided solid evidence of the mitochondrial outer membrane permeabilization. A previous study noticed that chrysin, enriched in CP, increased the expression of Bax in A375 cells [42]. In conclusion, these results provide strong evidence that a mitochondria-mediated intrinsic apoptosis and changes in the expression of Bax and Bcl-2 are induced by CP in A375 melanoma tumor cells. On the other hand, it has been reported that propolis was able to induced cell cycle arrest through the induction of Bcl-2/Bax regulation in a leukemic cell line [54].

Studies have highlightened that tumor cells with defective checkpoint functions are more vulnerable to anticancer agents [26]. Thus, the search for agents that interfere with cell cycle checkpoints has been a new direction toward enhancing the anti-tumor effect of tumor therapy. Our data suggested a G2/M phase arrest in CP-treated A375 cells. Previous studies also proved that propolis and its components deregulated the cell cycle in various tumor cells, suggesting that the cell cycle arrest may be universal in propolis-induced-apoptotic tumor cells [54,55,56,57]. In our study, the p-chk-1 was elevated, resulting in the suppression of the subsequent stream of cyclinB1/cdc-2. The inhibition of cdc-2 activation is believed to be involved in the DNA damage-induced G2 arrest [58], while the elevation of cdk-2-promoting cells entering the S phase is in consistent with our flow cytometry result. Another key protein regulating the cell-cycle arrest is p21 [59,60]. The p21 protein is synthesized in the G2 phase, promoting a pause in late G2 [61], and the G2 phase arrest can only be sustained when p21 is activated [62]. In our experiment, p21 was overexpressed after treatment of CP, and this contributes to the occurrence of G2/M arrest and DNA damage. In addition, p21 can disrupt the cell cycle in a p53-dependent or -independent manner [59,60,63]. Since p53 was found to be down regulated after CP treatment, we suggest that the up-regulation of p21 was mediated in a p53-independent manner.

The role that inflammation plays in tumor progression has been well studied [64]. The secretion of pro-inflammatory cytokines, including TNF, IL-1, and IL-8, is believed to build an inflammatory environment allowing tumor growth and metastasis [15]. When inflammation is activated, inflammasomes regulate caspase-1 activation and IL-1β secretion [15]. Studies have confirmed that IL-1β, IL-1α, IL-6, IL-8, IL-18 and their receptors are permanently expressed in malignant melanoma cells, suggesting that chronic inflammation exists during the melanoma development [65,66,67,68,69]. Propolis and its components have been well-known for inhibiting IL-1β secretion and for playing a role in NLRP3 and NLRC4 regulation [70]. Thus, we hypothesized that CP may exert its anti-inflammation effect on regulating melanoma progression and analyzed the expression of inflammation-related proteins by Western blot. We discovered that it is NLRP1, rather than NLRP3, was downregulated under CP treatment. In contrast to the NLRP1 protein level, its mRNA expression level is increased. Chai et al. also noticed that the mRNA level of *NLRP1* was not consistent with its protein level in human melanoma cell lines [10]. That may explain the variance between mRNA level and protein level of NLRP1 after CP treatment. NLRP1 and NLRP3 have been documented as activating the caspase-1 inflammasome, leading to IL-1β and IL-18 processing [71]. In our investigation, the mRNA level of *IL-1α*, *IL-1β*, and *IL-18* were all decreased, indicating the suppression of the inflammatory environment in human melanoma. Since NLRP1 is unique for containing a CARD binding motif, the activation of caspase-1 by NLRP1 does not require ASC involvement [13,72,73], and apoptotic caspases with a CARD domain, such as caspase-2 and caspase-9, are confirmed to be suppressed by NLRP1 [10]. Caspase-2 is as an important initiator caspase in intrinsic apoptosis pathways to guarantee cytochrome c release from mitochondria [74]. We found that caspase-2 was activated along with a decreased level of its full-length form after CP treatment, indicating that the intrinsic apoptosis induced by CP may partly be ascribed to the suppression of NLRP1 and the subsequent regulation of caspase-1, as well as caspase-2. Meanwhile, we discovered that another inflammatory caspase, caspase-4 [75], was downregulated, indicating that caspase-4 may also interact with NLRP1. Compared to recent studies indicating that NLRP1 is activated and able to promote tumor growth in human melanoma by enhancing inflammasome activity [9,10,15], we infer that it is NLRP1, rather than NLRP3, that is down-regulated under CP treatment, thereby attenuating the melanoma inflammatory microenvironment.

Furthermore, inflammation has been implied to associate with tumor metastasis. Thus, we also tested CP on the migration ability of A375 cells. In line with our expectation, the metastasis rate was slowed by CP in the wound healing assay. Meanwhile, the expression levels of MMP-2 and MMP-9 were also downregulated. Considering that chrysin has been reported to inhibit MMP-9 expression by suppressing ERK and JNK pathways, which are involved in the inflammation pathway in gastric cancer cells [76], we assume that the downregulation of the NLRP1-related inflammatory pathway also participates in the suppression of tumor metastasis in human melanoma cells induced by CP.

Autophagy was initially believed to be a tumor survival mechanism to help tumor cells against anticancer drugs; however, growing evidence suggests that autophagy can also promote apoptosis [23,77,78]. In our experiment, autophagy was activated by CP in human melanoma cells, which was confirmed by the decreasing LC3-I/LC3-II ratio and the accumulation of Atg5/Atg12, as well as p62. However, the expression of belcin1, which is reported to play a critical role in autophagosome formation, was down-regulated after CP treatment [79]. Taking into account that beclin1 interacts closely with inflammasome like NLRP-1, NLRP-3 and the Bcl-2 family, we inferred that the suppression of inflammation in CP-treated cells may have an influence on beclin1. On the other hand, there have been studies indicating that the induction of autophagy will reduce the caspase-1 dependent process of IL-1β secretion through an ROS-dependent mechanism [80]. Therefore, CP may be able to regulate the melanoma inflammatory microenvironment by activating autophagy. Then we applied CQ, an autophagy inhibitor, on CP-treated A375 cells. The apoptosis rate was dropped while comparing the co-incubation with CQ to CP treatment, suggesting that autophagy enhanced the cytotoxicity of CP on human melanoma. Therefore, CP may be able to regulate the melanoma inflammatory micro-environment by activating autophagy. A recent study has also reported that CP promoted autophagy in LPS-stimulated MDA-MB-231 cells [81], indicating that autophagy may be important in CP’s anti-proliferation effect on tumors. Further experiments need to be carried out to elucidate the precise mechanism.

## 5. Conclusions

In summary, the present study explored the in vitro anti-melanoma activity of CP. Here we firstly uncovered the pro-apoptosis effect and cell cycle arrest promotion of CP on human melanoma. The anti-inflammatory activity of CP by suppressing the NLRP1-related inflammatory pathway contributes to the suppression of melanoma progression. Also, autophagy was triggered after CP treatment, and inhibiting autophagy will weaken the cytotoxicity of CP against human melanoma cells. For a long time, people have paid much attention to the direct anti-tumor effect of propolis but ignored its combined effects with inflammation inhibition. We believe that this compelling evidence expands our understanding of the mechanism of the anti-melanoma ability of CP and provides a new approach for CP to be applied in melanoma therapy.

## Figures and Tables

**Table 1:** The primers used in quantitative RT-PCR. *GADPH* and *NLRP1* were obtained from Zhai et al. [10]. The *IL-1α*, *IL-1β* and *IL-18* were obtained from Primerbank [39].

NAME	Forward	Reverse
*GADPH*	CAGGGCTGCTTTTAACTCTGG	TGGGTGGAATCATATTGGAACA
*NLRP1*	GCTGGACCAGACAACTCTGA	GGTTTCCGTCTGCTGAAGAT
*IL-1α*	TGGTAGTAGCAACCAACGGGA	ACTTTGATTGAGGGCGTCATTC
*IL-1β*	AGCTACGAATCTCCGACCAC	CGTTATCCCATGTGTCGAAGAA
*IL-18*	TCTTCATTGACCAAGGAAATCGG	TCCGGGGTGCATTATCTCTAC

**Table 2:** The components identified in Chinese propolis (CP).

Compounds	RT(min)	[M + 1]^+^	Content (mg/g)
Caffeic acid	8.26	181.04226	8.24
p-Coumaric acid	10.36	165.04734	4.97
Ferulic acid	11.35	195.05791	2.58
Isoferulic acid	11.87	195.05791	3.16
Cinnamic acid	12.21	149.05243	0.62
Vanillic acid	15.04	169.04226
Kaempferol	16.26	287.04774	1.72
Myricetin	16.38	319.03757
Quercetin	16.39	303.04265	1.75
3,4-dimethoxycinnamic acid	17.02	209.07356	9.26
Apigenin	18.41	271.05282	3.61
Pinobanksin	18.82	273.06847	35.99
Luteotin	18.90	287.04774
Caffeic acid benzyl ester	23.61	271.08921
Chrysin	23.66	255.05791	50.99
Pinocembrin	24.22	257.07356	43.93
Galangin	24.44	271.05282	18.24
CAPE	24.74	285.10486	15.79
3-O-Acetyl pinobanksin	24.91	315.07904	73.81
Rutin	32.10	611.15339

## References

[1] Eberle J., Fecker L.F. (2017). Regulation of apoptosis in melanoma cells: Critical targets for therapeutic strategies. Melanoma Development.

[2] Godar D.E., Subramanian M., Merrill S.J. (2016). Cutaneous malignant melanoma incidences analyzed worldwide by skin type over advancing age of males and females: Evidence estrogen and androgenic hair are risk factors. J. Epidemiol. Res..

[3] Eggermont A.M., Spatz A., Robert C. (2014). Cutaneous melanoma. Lancet.

[4] GBD 2015 Disease and Injury Incidence and Prevalence Collaborators (2016). Global, regional, and national incidence, prevalence, and years lived with disability for 310 diseases and injuries, 1990–2015: A systematic analysis for the global burden of disease study 2015. Lancet.

[5] Martinon F. (2012). Dangerous liaisons: Mitochondrial DNA meets the nlrp3 inflammasome. Immunity.

[6] Awad F., Assrawi E., Louvrier C., Jumeau C., Giurgea I., Amselem S., Karabina S.-A. (2018). Photoaging and skin cancer: Is the inflammasome the missing link?. Mech Ageing Dev..

[7] Nasti T.H., Timares L. (2012). Inflammasome activation of IL-1 family mediators in response to cutaneous photodamage. Photochem. Photobiol..

[8] Meyer C., Sevko A., Ramacher M., Bazhin A.V., Falk C.S., Osen W., Borrello I., Kato M., Schadendorf D., Baniyash M. (2011). Chronic inflammation promotes myeloid-derived suppressor cell activation blocking antitumor immunity in transgenic mouse melanoma model. Proc. Natl. Acad. Sci. USA.

[9] Melnikova V.O., Bar-Eli M. (2009). Inflammation and melanoma metastasis. Pigment Cell Melanoma Res..

[10] Zhai Z., Liu W., Kaur M., Luo Y., Domenico J., Samson J.M., Shellman Y.G., Norris D.A., Dinarello C.A., Spritz R.A. (2017). Nlrp1 promotes tumor growth by enhancing inflammasome activation and suppressing apoptosis in metastatic melanoma. Oncogene.

[11] Nikolova P.N., Pawelec G.P., Mihailova S.M., Ivanova M.I., Myhailova A.P., Baltadjieva D.N., Marinova D.I., Ivanova S.S., Naumova E.J. (2007). Association of cytokine gene polymorphisms with malignant melanoma in caucasian population. Cancer Immunol. Immunother..

[12] Qin Y., Ekmekcioglu S., Liu P., Duncan L.M., Lizée G., Poindexter N., Grimm E.A. (2011). Constitutive aberrant endogenous interleukin-1 facilitates inflammation and growth in human melanoma. Mol. Cancer Res..

[13] Allen I.C. (2014). Non-inflammasome forming nlrs in inflammation and tumorigenesis. Front. Immunol..

[14] Kutikhin A.G., Yuzhalin A.E. (2012). Inherited variation in pattern recognition receptors and cancer: Dangerous liaisons?. Cancer Manag. Res..

[15] Dunn J.H., Ellis L.Z., Fujita M. (2012). Inflammasomes as molecular mediators of inflammation and cancer: Potential role in melanoma. Cancer Lett..

[16] Debnath J., Baehrecke E.H., Kroemer G. (2005). Does autophagy contribute to cell death?. Autophagy.

[17] Mizushima N., Komatsu M. (2011). Autophagy: Renovation of cells and tissues. Cell.

[18] Marino M.L., Pellegrini P., Di Lernia G., Djavaheri-Mergny M., Brnjic S., Zhang X., Hägg M., Linder S., Fais S., Codogno P. (2012). Autophagy is a protective mechanism for human melanoma cells under acidic stress. J. Biol. Chem..

[19] Jounai N., Kobiyama K., Shiina M., Ogata K., Ishii K.J., Takeshita F. (2011). Nlrp4 negatively regulates autophagic processes through an association with beclin1. J. Immunol..

[20] Deretic V. (2012). Autophagy as an innate immunity paradigm: Expanding the scope and repertoire of pattern recognition receptors. Curr. Opin. Immunol..

[21] Qian S., Fan J., Billiar T.R., Scott M.J. (2017). Inflammasome and autophagy regulation: A two-way street. Mol. Med..

[22] Marcilla-Etxenike A., Martín M.L., Noguera-Salvà M.A., García-Verdugo J.M., Soriano-Navarro M., Dey I., Escribá P.V., Busquets X. (2012). 2-hydroxyoleic acid induces er stress and autophagy in various human glioma cell lines. PLoS ONE.

[23] Yeh P.-S., Wang W., Chang Y.-A., Lin C.-J., Wang J.-J., Chen R.-M. (2016). Honokiol induces autophagy of neuroblastoma cells through activating the pi3k/akt/mtor and endoplasmic reticular stress/erk1/2 signaling pathways and suppressing cell migration. Cancer Lett..

[24] Li H., Zhang J., Sun L., Li B., Gao H., Xie T., Zhang N., Ye Z. (2015). Celastrol induces apoptosis and autophagy via the ros/jnk signaling pathway in human osteosarcoma cells: An in vitro and in vivo study. Cell Death Dis..

[25] Stewart Z.A., Westfall M.D., Pietenpol J.A. (2003). Cell-cycle dysregulation and anticancer therapy. Trends Pharmacol. Sci..

[26] Pietenpol J., Stewart Z. (2002). Cell cycle checkpoint signaling: Cell cycle arrest versus apoptosis. Toxicology.

[27] Blagosklonny M.V., Pardee A.B. (2002). The restriction point of the cell cycle. Cell Cycle.

[28] Gismondi A., Trionfera E., Canuti L., Di Marco G., Canini A. (2017). Royal jelly lipophilic fraction induces antiproliferative effects on sh-sy5y human neuroblastoma cells. Oncol. Rep..

[29] Ramadan M.F., Al-Ghamdi A. (2012). Bioactive compounds and health-promoting properties of royal jelly: A review. J. Funct. Foods.

[30] Sforcin J. (2007). Propolis and the immune system: A review. J. Ethnopharmacol..

[31] Sforcin J.M., Bankova V. (2011). Propolis: Is there a potential for the development of new drugs?. J. Ethnopharmacol..

[32] Osés S., Pascual-Maté A., Fernández-Muiño M., López-Díaz T., Sancho M. (2016). Bioactive properties of honey with propolis. Food Chem..

[33] Xuan H., Li Z., Yan H., Sang Q., Wang K., He Q., Wang Y., Hu F. (2014). Antitumor activity of Chinese propolis in human breast cancer mcf-7 and mda-mb-231 cells. Evid.-Based Complement. Altern. Med..

[34] Ishihara M., Naoi K., Hashita M., Itoh Y., Suzui M. (2009). Growth inhibitory activity of ethanol extracts of Chinese and brazilian propolis in four human colon carcinoma cell lines. Oncol. Rep..

[35] Zhao Y., Tian W., Peng W. (2014). Anti-proliferation and insulin resistance alleviation of hepatocellular carcinoma cells hepg2 in vitro by Chinese propolis. J. Food Nutr. Res..

[36] Patel S. (2016). Emerging adjuvant therapy for cancer: Propolis and its constituents. J. Diet. Suppl..

[37] Sha N., Guan S.-H., Lu Z.-Q., Chen G.-T., Huang H.-L., Xie F.-B., Yue Q.-X., Liu X., Guo D.-A. (2009). Cytotoxic constituents of Chinese propolis. J. Nat. Product..

[38] Zheng Y., Wang K., Wu Y., Chen Y., Chen X., Hu C.W., Hu F. (2018). Pinocembrin induces er stress mediated apoptosis and suppresses autophagy in melanoma cells. Cancer Lett..

[39] Wang X., Spandidos A., Wang H., Seed B. (2011). Primerbank: A pcr primer database for quantitative gene expression analysis, 2012 update. Nucleic Acids Res..

[40] Jin X.-L., Wang K., Li Q.-Q., Tian W.-L., Xue X.-F., Wu L.-M., Hu F.-L. (2017). Antioxidant and anti-inflammatory effects of Chinese propolis during palmitic acid-induced lipotoxicity in cultured hepatocytes. J. Funct. Foods.

[41] Zhang C., Huang S., Wei W., Ping S., Shen X., Li Y., Hu F. (2014). Development of high-performance liquid chromatographic for quality and authenticity control of Chinese propolis. J. Food Sci..

[42] Pichichero E., Cicconi R., Mattei M., Canini A. (2011). Chrysin-induced apoptosis is mediated through p38 and bax activation in b16-f1 and a375 melanoma cells. Int. J. Oncol..

[43] Biggar K.K., Storey K.B. (2009). Perspectives in cell cycle regulation: Lessons from an anoxic vertebrate. Curr. Genom..

[44] Fitzgerald K.A. (2010). Nlr-containing inflammasomes: Central mediators of host defense and inflammation. Eur. J. Immunol..

[45] Lazova R., Klump V., Pawelek J. (2010). Autophagy in cutaneous malignant melanoma. J. Cutan. Pathol..

[46] Usia T., Banskota A.H., Tezuka Y., Midorikawa K., Matsushige K., Kadota S. (2002). Constituents of Chinese propolis and their antiproliferative activities. J. Nat. Prod..

[47] Xuan H., Wang Y., Li A., Fu C., Wang Y., Peng W. (2016). Bioactive components of Chinese propolis water extract on antitumor activity and quality control. Evid.-Based Complement. Altern. Med..

[48] Li Y., Chen L., Jiang F., Yang Y., Wang X., Zhang Z., Li Z., Li L. (2015). Caffeic acid improves cell viability and protects against DNA damage: Involvement of reactive oxygen species and extracellular signal-regulated kinase. Braz. J. Med. Biol. Res..

[49] Demestre M., Messerli S., Celli N., Shahhossini M., Kluwe L., Mautner V., Maruta H. (2009). Cape (caffeic acid phenethyl ester)-based propolis extract (bio 30) suppresses the growth of human neurofibromatosis (nf) tumor xenografts in mice. Phytother Res..

[50] Wu J., Omene C., Karkoszka J., Bosland M., Eckard J., Klein C.B., Frenkel K. (2011). Caffeic acid phenethyl ester (cape), derived from a honeybee product propolis, exhibits a diversity of anti-tumor effects in pre-clinical models of human breast cancer. Cancer Lett..

[51] Angelo G., Lorena C., Marta G., Antonella C. (2014). Biochemical composition and antioxidant properties of lavandula angustifolia miller essential oil are shielded by propolis against uv radiations. Photochem. Photobiol..

[52] Ahn M.-R., Kumazawa S., Usui Y., Nakamura J., Matsuka M., Zhu F., Nakayama T. (2007). Antioxidant activity and constituents of propolis collected in various areas of China. Food Chem..

[53] Taira N., Nguyen B.C.Q., Be Tu P.T., Tawata S. (2016). Effect of Okinawa propolis on pak1 activity, caenorhabditis elegans longevity, melanogenesis, and growth of cancer cells. J. Agric. Food Chem..

[54] Motomura M., Kwon K.M., Suh S.-J., Lee Y.-C., Kim Y.-K., Lee I.-S., Kim M.-S., Kwon D.Y., Suzuki I., Kim C.-H. (2008). Propolis induces cell cycle arrest and apoptosis in human leukemic u937 cells through bcl-2/bax regulation. Environ. Toxicol. Pharmacol..

[55] Chen C.-N., Weng M.-S., Wu C.-L., Lin J.-K. (2004). Comparison of radical scavenging activity, cytotoxic effects and apoptosis induction in human melanoma cells by Taiwanese propolis from different sources. Evid.-Based Complement. Altern. Med..

[56] Sawicka D., Car H., Borawska M.H., Nikliński J. (2012). The anticancer activity of propolis. Folia Histochem. Cytobiol..

[57] Zhang Q., Zhao X.-H., Wang Z.-J. (2008). Flavones and flavonols exert cytotoxic effects on a human oesophageal adenocarcinoma cell line (oe33) by causing g2/m arrest and inducing apoptosis. Food Chem. Toxicol..

[58] Hwang A., Muschel R.J. (1998). Radiation and the g2 phase of the cell cycle. Radiat. Res..

[59] Cayrol C., Knibiehler M., Ducommun B. (1998). P21 binding to pcna causes g1 and g2 cell cycle arrest in p53-deficient cells. Oncogene.

[60] Waldman T., Kinzler K.W., Vogelstein B. (1995). P21 is necessary for the p53-mediated g1 arrest in human cancer cells. Cancer Res..

[61] Bates S., Ryan K.M., Phillips A.C., Vousden K.H. (1998). Cell cycle arrest and DNA endoreduplication following p21 waf1/cip1 expression. Oncogene.

[62] Bunz F., Dutriaux A., Lengauer C., Waldman T., Zhou S., Brown J., Sedivy J., Kinzler K., Vogelstein B. (1998). Requirement for p53 and p21 to sustain g2 arrest after DNA damage. Science.

[63] Ropponen K., Kellokoski J., Lipponen P., Pietiläinen T., Eskelinen M., Alhava E., Kosma V. (1999). P21/waf1 expression in human colorectal carcinoma: Association with p53, transcription factor ap-2 and prognosis. Br. J. Cancer.

[64] Philip M., Rowley D.A., Schreiber H. (2004). Inflammation as a Tumor Promoter in Cancer Induction, Seminars in Cancer Biology.

[65] Voronov E., Shouval D.S., Krelin Y., Cagnano E., Benharroch D., Iwakura Y., Dinarello C.A., Apte R.N. (2003). IL-1 is required for tumor invasiveness and angiogenesis. Proc. Natl. Acad. Sci. USA.

[66] Colombo M., Maccalli C., Mattei S., Melani C., Radrizzani M., Parmiani G. (1992). Expression of cytokine genes, including IL-6, in human malignant melanoma cell lines. Melanoma Res..

[67] Schadendorf D., Möller A., Algermissen B., Worm M., Sticherling M., Czarnetzki B. (1993). IL-8 produced by human malignant melanoma cells in vitro is an essential autocrine growth factor. J. Immunol..

[68] Park H., Byun D., Kim T.S., Kim Y.I., Kang J.S., Hahm E.S., Kim S.H., Lee W.J., Song H.K., Yoon D.Y. (2001). Enhanced il-18 expression in common skin tumors. Immunol. Lett..

[69] Vidal-Vanaclocha F., Fantuzzi G., Mendoza L., Fuentes A.M., Anasagasti M.J., Martín J., Carrascal T., Walsh P., Reznikov L.L., Kim S.-H. (2000). Il-18 regulates il-1β-dependent hepatic melanoma metastasis via vascular cell adhesion molecule-1. Proc. Natl. Acad. Sci. USA.

[70] Tőzsér J., Benkő S. (2016). Natural compounds as regulators of nlrp3 inflammasome-mediated il-1β production. Mediat. Inflamm..

[71] Fritz J.H., Ferrero R.L., Philpott D.J., Girardin S.E. (2006). Nod-like proteins in immunity, inflammation and disease. Nat. Immunol..

[72] Damiano J.S., Reed J.C. (2004). Card proteins as therapeutic targets in cancer. Curr. Drug Targets.

[73] Liu W., Luo Y., Dunn J.H., Norris D.A., Dinarello C.A., Fujita M. (2013). Dual role of apoptosis-associated speck-like protein containing a card (asc) in tumorigenesis of human melanoma. J. Investig. Dermatol..

[74] Lassus P., Opitz-Araya X., Lazebnik Y. (2002). Requirement for caspase-2 in stress-induced apoptosis before mitochondrial permeabilization. Science.

[75] Martinon F., Tschopp J. (2007). Inflammatory caspases and inflammasomes: Master switches of inflammation. Cell Death Differ..

[76] Xia Y., Lian S., Khoi P.N., Yoon H.J., Joo Y.E., Chay K.O., Kim K.K., Do Jung Y. (2015). Chrysin inhibits tumor promoter-induced mmp-9 expression by blocking ap-1 via suppression of erk and jnk pathways in gastric cancer cells. PLoS ONE.

[77] Saiki S., Sasazawa Y., Imamichi Y., Kawajiri S., Fujimaki T., Tanida I., Kobayashi H., Sato F., Sato S., Ishikawa K.-I. (2011). Caffeine induces apoptosis by enhancement of autophagy via pi3k/akt/mtor/p70s6k inhibition. Autophagy.

[78] Denton D., Nicolson S., Kumar S. (2012). Cell death by autophagy: Facts and apparent artefacts. Cell Death Differ..

[79] Kang R., Zeh H., Lotze M., Tang D. (2011). The beclin 1 network regulates autophagy and apoptosis. Cell Death Differ..

[80] Harris J., Hartman M., Roche C., Zeng S.G., O’Shea A., Sharp F.A., Lambe E.M., Creagh E.M., Golenbock D.T., Tschopp J. (2011). Autophagy controls il-1β secretion by targeting pro-il-1β for degradation. J. Biol. Chem..

[81] Chang H., Wang Y., Yin X., Liu X., Xuan H. (2017). Ethanol extract of propolis and its constituent caffeic acid phenethyl ester inhibit breast cancer cells proliferation in inflammatory microenvironment by inhibiting tlr4 signal pathway and inducing apoptosis and autophagy. BMC Complement. Altern. Med..

